# An In Vitro System to Study the Effect of Subchondral Bone Health on Articular Cartilage Repair in Humans

**DOI:** 10.3390/cells10081903

**Published:** 2021-07-27

**Authors:** Timothy Hopkins, Karina T. Wright, Nicola J. Kuiper, Sally Roberts, Paul Jermin, Peter Gallacher, Jan Herman Kuiper

**Affiliations:** 1 School of Pharmacy and Bioengineering, Keele University, Staffordshire ST5 5BG, UK; karina.wright1@nhs.net (K.T.W.); n.j.kuiper@keele.ac.uk (N.J.K.); sally.roberts4@nhs.net (S.R.); p.jermin@nhs.net (P.J.); peter.gallacher1@nhs.net (P.G.); j.h.kuiper@keele.ac.uk (J.H.K.); 2 Robert Jones and Agnes Hunt Orthopaedic Hospital, Shropshire SY10 7AG, UK

**Keywords:** cartilage repair, human, osteoarthritis, chondrocytes, bone-marrow-derived mesenchymal stromal cells (BM-MSCs), co-culture, in vitro modelling

## Abstract

Chondrocyte-based cartilage repair strategies, such as articular chondrocyte implantation, are widely used, but few studies addressed the communication between native subchondral bone cells and the transplanted chondrocytes. An indirect co-culture model was developed, representing a chondrocyte/scaffold-construct repair of a cartilage defect adjoining bone, where the bone could have varying degrees of degeneration. Human BM-MSCs were isolated from two areas of subchondral bone in each of five osteochondral tissue specimens from five patients undergoing knee arthroplasty. These two areas underlaid the macroscopically and histologically best and worst cartilage, representing early and late-stage OA, respectively. BM-MSCs were co-cultured with normal chondrocytes suspended in agarose, with the two cell types separated by a porous membrane. After 0, 7, 14 and 21 days, chondrocyte–agarose scaffolds were assessed by gene expression and biochemical analyses, and the abundance of selected proteins in conditioned media was assessed by ELISA. Co-culture with late-OA BM-MSCs resulted in a reduction in GAG deposition and a decreased expression of genes encoding matrix-specific proteins (*COL2A1* and *ACAN*), compared to culturing with early OA BM-MSCs. The concentration of TGF-β1 was significantly higher in the early OA conditioned media. The results of this study have clinical implications for cartilage repair, suggesting that the health of the subchondral bone may influence the outcomes of chondrocyte-based repair strategies.

## 1. Introduction

In the human knee, the articular cartilage (AC) forms a complex, biomechanically functional unit with the subchondral bone (SB), the osteochondral unit, in which the components act together to dissipate forces generated during articulation [1]. The existence of biochemical communication between the AC and SB was initially considered unlikely, due to the perceived impenetrability of the calcified cartilage to even small molecules [2,3,4]. However, the presence of certain physical features at the osteochondral junction, as well as common biochemical pathways that are able to activate cellular process in both tissues, suggested that the two were not as separate as previously thought [2,4,5,6,7,8]. Subsequently, several groups used ex vivo models of the osteochondral junction to demonstrate that communication through the calcified cartilage was possible via the diffusion of both small and large molecules [5,9,10,11]. As such, the osteochondral unit can be considered a biochemically and biomechanically functional unit, and processes that disrupt homeostasis in either tissue will therefore alter the properties and function of the other [1,3,4].

This is reflected in osteoarthritis (OA) pathogenesis, in which SB thickening and AC degradation are two of the most common features, and pathological changes in the SB often parallel those seen in the overlying AC [12,13]. However, little consideration is given to the health status of the SB in influencing the outcomes of cell-based strategies for AC repair, which are widely used in the treatment of early degenerative or traumatic lesions. The goal of cell-based tissue engineering strategies is to provide the optimal cell type with the optimal environment to be able to produce cartilaginous tissue that closely resembles the structure and composition of native tissue [14]. As the SB is not routinely addressed in any significant way by cell therapy, other than restoration of joint mechanics, any altered biochemical signalling that existed between the cells of the SB and the degenerated AC, will likely continue to act on cells that are implanted as the cell therapy and could influence the outcome.

Finally, the majority of AC repair techniques are indicated for traumatic lesions, or early degenerative lesions, and although one cell-based option has shown promising results for the treatment of OA [15] (Invossa), the efficacy of traditional cartilage repair techniques is demonstrably lower in patients with OA [16]. One of the difficulties in treating cartilage degeneration in end-stage OA is determining the contribution of the other tissues of the joint to the pathogenic changes in the AC, given that end-stage OA represents an ‘organ-level’ failure in which all joint tissues are involved. The SB is of particular interest and determining the contribution of the SB in AC repair at the various stages of OA is important for the future development of cell therapies for OA. 

While some authors have stressed the biomechanical importance of the SB in AC repair, there is, to our knowledge, no published work investigating the influence of cellular crosstalk from the SB on the AC repair tissue [17,18]. However, several in vitro studies have investigated the ability of cells from the subchondral bone to modulate the behaviour of mature chondrocytes, in OA progression models. Westacott and colleagues demonstrated that the indirect co-culture of cartilage explants with primary osteoblasts taken from OA patients led to a significant increase in glycosaminoglycan (GAG) secretion into conditioned media, compared to those cultured with osteoblasts from non-arthritic patients [19]. A similar model was used by Sanchez and colleagues, in two studies published in 2005, to show that soluble factors secreted by human osteoblasts can modulate the phenotype of human OA chondrocytes. The authors reported a differential effect on chondrocyte phenotype when they were cultured with osteoblasts isolated from normal or sclerotic regions of the subchondral bone [20,21]. The focus of the above in vitro studies was the influence of the interaction between SB and AC cells on the progression of OA, rather than its influence on AC repair—the remit of the present study. For this reason, the cartilage cells or explanted tissues used in these earlier studies are not representative of those that may be implanted for AC repair. 

Here we present a novel, in vitro, co-culture model of the osteochondral unit, developed to investigate the effect of SB health on a cell-based cartilage repair technique, in early and late-stage OA. 

## 2. Materials and Methods

### 2.1. Sample Collection and Grading

Knee condylar samples of osteochondral tissue were collected from 5 patients (2 males and 3 females; age, 72.8 ± 2.2 y/o) undergoing total knee replacement (TKR) surgery at the RJAH Orthopaedic Hospital NHS Trust and who had provided written, informed consent. A tibial plateau from a young patient (male; 22 y/o) with no medical history of OA, and who had sadly passed away as the result of a road traffic collision, was obtained through the NHS Blood and Transplant service. Favourable ethical approval was given by the National Research Ethics Service (11/NW/0875), and all experiments were performed in accordance with the relevant guidelines and regulations.

The AC on excised osteochondral tissue was initially graded macroscopically, using the International Cartilage Repair Society (ICRS) Cartilage Lesion Classification System, which consists of 5 grades: grade 0, ‘normal cartilage’; grade 1, ‘nearly normal cartilage’; grade 2, ‘abnormal cartilage’; grade 3, ‘severely abnormal cartilage—without SB involvement’; and grade 4, ‘severely abnormal cartilage—with SB involvement’ [22]. Osteochondral tissue samples were also graded histologically. The AC was graded by using the Osteoarthritis Research Society International (OARSI) OA Cartilage Histopathology Assessment System, which comprises 7 grades: grade 0, ‘normal AC’; grade 1, ‘surface intact’; grade 2, ‘surface discontinuity’; grade 3, ‘vertical fissures’; grade 4, ‘erosion’; grade 5, ‘denudation’; and grade 6, ‘deformation’ [23]. The SB was graded by using the Subchondral Bone Histological Grading System, which consists of 4 grades: grade 0, ‘the early stages of OA’; grade 1, ’some subchondral bone sclerosis’; grade 2, ‘distinct increase in SB sclerosis and bone volume’; and grade 3, ‘late stage disease’ [24]. The osteochondral tissue that demonstrated the best and worst macroscopic and histological scores, for each patient, served as within-donor, paired specimens with varying degrees of degeneration, representing early and late-stage OA. These regions, and the isolated cells, are henceforth referred to as ‘early’ and ‘late’ for simplicity. 

### 2.2. Cell Isolation and Expansion 

Bone-marrow-derived mesenchymal stromal cells (BM-MSCs) were isolated from the SB of the early and late osteochondral tissue, using an adapted protocol for the extraction of BM-MSCs from bone-marrow aspirate. In brief, the SB was disrupted by using sterile scissor forceps to release bone marrow and bone chips, before being added to a sterile sample pot containing 30 mL of complete culture media (Dulbecco’s modified eagle medium: nutrient mixture F-12 (DMEM/F12; 1:1) (Life Technologies, Paisley, UK) supplemented with 10% (*v*/*v*) foetal bovine serum (FBS; Life Technologies) and 1% (*v*/*v*) penicillin–streptomycin (P/S; 5000 U/mL; Gibco, Loughborough, UK). The pot was shaken vigorously for 5 min to create a pseudo-bone-marrow aspirate, which was then split between two sterile 50 mL tubes by carefully layering 15 mL aspirate onto 10 mL Lymphoprep™ (STEMCELL technologies, Cambridge, UK) in each. The tubes were centrifuged (900× *g* for 20 min) and the mononuclear cells were harvested by collecting the resulting buffy coat layer, which was added to 10 mL complete culture medium and centrifuged again (750× *g* for 10 min). The resulting cell pellet was resuspended in complete culture medium, a viable cell count was performed by trypan blue exclusion and the cells were seeded at a density of 20 × 10^6^ cells per 75 cm^2^ tissue culture flask in BM-MSC seeding media (DMEM/F12; 1:1 supplemented with 20% (*v*/*v*) FBS and 1% (*v*/*v*) P/S; (5000 U/mL). After 24 h, non-adherent cells were removed by PBS wash and media change and adherent cells were cultured in monolayer and maintained in a humidified atmosphere, at 37 °C, 5% CO_2_, in complete culture media. 

Chondrocytes were isolated from a sample of cartilage taken from the knee of a patient with no history of OA. AC was excised from the tibial plateau of the donated knee, and chondrocytes were extracted as previously described [25]. In brief, the cartilage was weighed and minced into small pieces (roughly 1 mm^3^) with a sterile scalpel and digested in type II collagenase (245 IU/mL; Worthington Biochemical Corp, Lakewood, NJ, USA) in DMEM/F12 (1:1) containing 1% P/S (5000 U/mL) for 16 h at 37 °C (10 mL type II collagenase was used per 200 mg of cartilage). After 16 h, the resulting suspension was passed through a sterile 40 μm cell strainer (ThermoFisher Scientific, Loughborough, UK) and centrifuged (350× *g* for 10 min) to produce a cell pellet. The cell pellet was then resuspended in chondrocyte culture media (DMEM/F12 (1:1) supplemented with 10% (*v*/*v*) FBS, 1% (*v*/*v*) P/S (5000 U/mL) and 0.5% Ascorbic Acid (stock, 12.5 mg/mL; final, 62 μg/mL; Sigma-Aldrich, Dorset, UK). Chondrocytes were seeded at a density of 5 × 10^3^ cells per cm^2^ and were maintained in monolayer in a humidified atmosphere at 37 °C and 5% CO_2_ in chondrocyte culture media.

All cell populations were maintained in monolayer culture until 80% confluent, at which point they were passaged by incubation with trypsin–ethylenediamine tetraacetic acid (trypsin–EDTA; 0.05% (*w*/*v*), Gibco), and a viable cell count was performed by trypan blue exclusion. Cells were then either reseeded at a density of 5 × 10^3^ cells/cm^2^ for subculture or cryopreserved in 10% (*v*/*v*) dimethyl sulfoxide (DMSO; Sigma-Aldrich) in FBS, at a density of 1 × 10^6^ cells per mL.

### 2.3. Co-Culture Model

**Figure 1:**
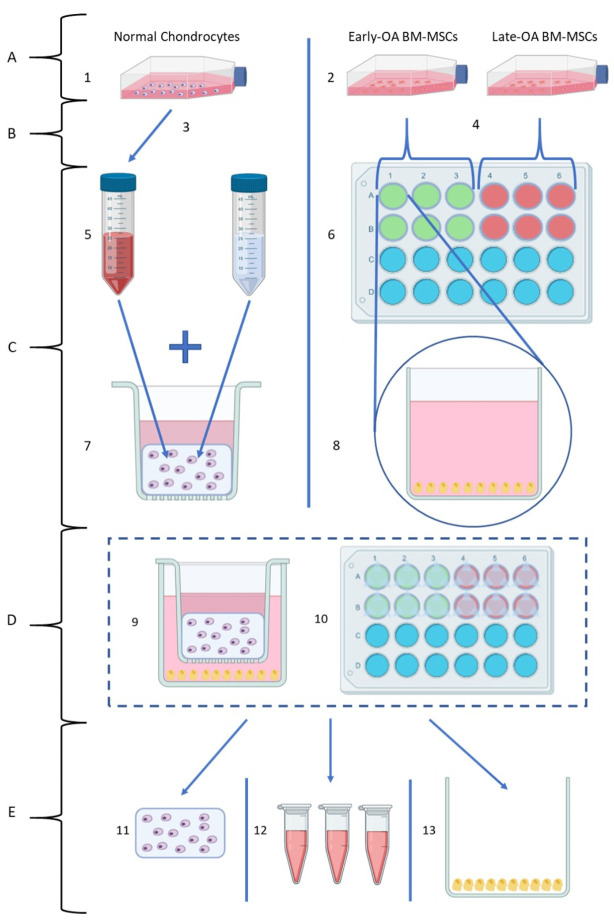
Overview of the co-culture model (created using Biorender.com). (**A**) Recovery of cells from long-term storage: (1) Chondrocytes were recovered from liquid nitrogen storage and cultured in monolayer. (2) Donor-matched BM-MSC populations recovered from liquid nitrogen storage and cultured in monolayer. (**B**) Cell Collection: (3) Once 80% confluent, chondrocytes were collected by trypsinisation, and the required number for the model was separated from the suspension. (4) Once 80% confluent, BM-MSCs were collected by trypsinisation and counted, and the required number for the model was separated from the suspension. (**C**) Individual cell preparation for co-culture model: (5) Chondrocyte suspension was combined with 4% agarose solution to form a 1:1 mixture. (6) Donor-matched early OA and late-OA BM-MSCs seeded in 24-well plates (top view). (7) Agarose–chondrocyte mixture pipetted into 24-well plate transwell inserts. (8) BM-MSCs in 24-well plate (side-view) with osteogenic differentiation media added. (**D**) Co-culture setup: (9) Co-culture model (individual well, side view). (10) Co-culture model (full plate, top view). (**E**) Harvest of the co-culture model for analysis at endpoints (day 0, day 7, day 14 and day 21): (11) Chondrocyte–agarose scaffolds were removed for gene expression, histological and biochemical analyses. (12) Conditioned medium was removed for analysis of secreted protein abundance by ELISA. (13) BM-MSCs removed for gene expression and biochemical analyses (data not shown).

A novel in vitro model was established, in which donor-matched early and late BM-MCSs were indirectly co-cultured with healthy, allogeneic chondrocytes. Throughout the experiment, BM-MSCs were maintained in monolayer in 24-well plates (Sarstedt, Leicester, UK) and chondrocytes were encapsulated in agarose within transwell inserts (TC-Insert, 24-well, 0.4 µm pore; Sarstedt), which were then placed into the BM-MSC-containing wells. In this way, the two cell types were indirectly co-cultured, with no direct contact between the cells; however, communication by soluble factors was permitted. An overview of the co-culture model is shown in Figure 1. 

#### 2.3.1. Patients and Cell Populations 

The co-culture model was performed with donor-matched populations of early and late BM-MSCs, isolated from the two osteochondral specimens, from each patient, that showed the largest difference in SB histological grade. Culture-expanded chondrocytes isolated from the donated knee of the healthy donor were used in the co-culture models of all five patients. Due to the complexity of the model and the labour-intensive setup and endpoint days, the patients were split into three batches that were staggered in their setup by two weeks, with two patients in the first two batches, and the final patient in the third batch. Each batch ran for approximately 50 days in total, dictated by the speed of proliferation of the various cell populations in culture.

#### 2.3.2. Model Setup

**Figure 2:**
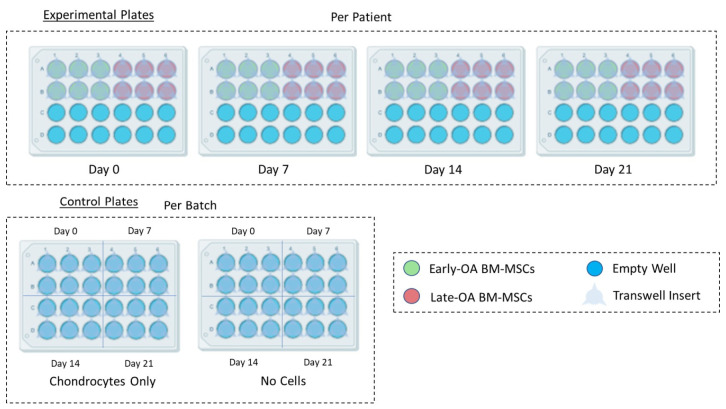
Plate layout for the setup of the co-culture model (created using Biorender.com). For each patient, four experimental plates (day 0, day 7, day 14 and day 21) were seeded with both early OA and late-OA BM-MSCs (6 wells each). BM-MSCs were osteogenically differentiated, and then chondrocyte-seeded agarose scaffolds in transwell inserts were added to the experimental plates and to the chondrocyte-only control plate. Inserts containing agarose alone, with no chondrocytes, were added to the no cells control plate. Experimental plates were set up per patient, whereas the chondrocyte only and no cell control plates were set up per batch.

Donor-matched BM-MSC populations were recovered from liquid nitrogen storage and seeded at a density 5 × 10^3^ cells per cm^2^ in 24-well plates as required (Figure 2). Once the BM-MSC populations reached 80% confluence in the 24-well plates, osteogenic differentiation media (DMEM/F12 (1:1) supplemented with 10% (*v*/*v*) FBS and 1% (*v*/*v*) P/S (5000 U/mL), L-Ascorbic Acid-2-phosphate (50 µM), β-glycerophosphate (10 mM) and dexamethasone (100 nM) (all Sigma-Aldrich; [26]) was added to the BM-MSCs and the cells were allowed to differentiate for three weeks, with media exchanged three times per week. 

Chondrocytes were recovered from liquid nitrogen storage as required and cultured in monolayer in tissue culture flasks until they reached 80% confluence, at which point they were collected by trypsinisation and counted by trypan blue exclusion. A 2x agarose solution was prepared by dissolving low gelling temperature (Type VII-A) agarose (4% (*w*/*v*); Sigma-Aldrich) in PBS. The solution was sterilised by autoclaving and then maintained at 37 °C on a heat block, with constant stirring. The required number of chondrocytes for each batch was calculated and separated from the cell suspension, following trypsinisation and centrifuged (179× *g* for 10 min). A chondrocyte suspension with a concentration of 2 × 10^6^ chondrocytes per mL was prepared by re-suspending the cell pellet with the required volume of 2x chondrocyte culture media: DMEM/F12 (1:1) supplemented with 20% (*v*/*v*) FBS, 2% (*v*/*v*) P/S (5000 U/mL) and 1% Ascorbic Acid (final concentration: 124 μg/mL). The cell suspension was then quickly combined 1:1 with an equal volume of the 4% (*w*/*v*) agarose in PBS to create a final mixture of 2% (*w*/*v*) agarose that contained 1 × 10^6^ chondrocytes per mL in 1x chondrocyte culture media. The mixture was maintained at 37 °C to prevent the agarose from setting and was stirred slowly throughout, using a magnetic stirrer to prevent separation without introducing bubbles. Using a reverse pipetting technique, 200 µL of the chondrocyte–agarose mixture was carefully pipetted into 24-well plate transwell inserts (TC-Insert, 24 well, 0.4 µm pore; Sarstedt) on sterile Petri dishes (Sarstedt). The agarose-containing inserts were allowed to set for 15 min at room temperature. 

Inserts were randomly assigned into the experimental and chondrocyte-only control plates (Figure 2). Then 250 µL of chondrogenic differentiation media (DMEM/F12 (1:1) supplemented with 2% (*v*/*v*) FBS, 1% (*v*/*v*) P/S (5000 U/mL) and 1% (*v*/*v*) insulin–transferrin–selenium–ethanolamine (ITS-X; Gibco) and freshly supplemented at time of use with L-Ascorbic Acid-2-phosphate (1 mM), dexamethasone (100 nM) and sodium pyruvate (1 mM; all Sigma-Aldrich) and transforming growth factor-β1 (TGF-β1; PeproTech, London, UK; 10 ng/mL) [27]) was added on top of the agarose in the insert. Then 1.5 mL of osteogenic differentiation media was carefully added to the bottom of the well, avoiding mixing between the two media types. 

**Figure 3:**
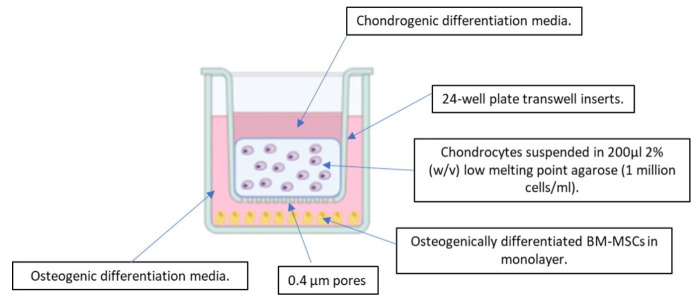
Schematic of the indirect co-culture model (created using Biorender.com). Side view of a single well of an experimental plate.

The resulting co-culture system consisted of osteogenically differentiated BM-MSCs, isolated from early OA and late-OA SB, indirectly co-cultured with healthy chondrocytes. BM-MSC populations were maintained in monolayer, while agarose-encapsulated chondrocytes were suspended above the BM-MSCs within transwell inserts (Figure 3). Both media types were exchanged every 2 to 3 days in all experimental and control plates. 

#### 2.3.3. Controls 

Chondrocyte-only controls were established by adding transwell inserts containing chondrocyte–agarose scaffolds to empty 24-well plates, rather than those seeded with BM-MSCs. Chondrocyte-only controls were set up per batch, rather than per patient, as the same chondrocytes were used for all patients. 

No-cell controls consisted of transwell inserts containing 200 µL of 2% (*w*/*v*) low gelling temperature (Type VII-A) agarose in chondrocyte culture media (4% (*w*/*v*) agarose in PBS mixed 1:1 with 2x chondrogenic media) placed into empty 24-well plates. The no-cell control was established to act as an assay blank for each timepoint and was set up per batch, rather than per patient. Both controls received the same media types and volumes as the experimental plates, and they were exchanged every 2 to 3 days.

#### 2.3.4. Endpoint Analysis—Chondrocyte–Agarose Scaffolds

At each endpoint, the transwell inserts containing the agarose scaffolds were carefully removed from the 24-well plates, using sterile forceps and transferred into sterile Petri dishes. The agarose scaffolds were removed from the transwell inserts, using a 60 mm Biopsy Punch (Integra™, Miltex™, ThermoFisher Scientific) and transferred into a sterile Petri dish. The scaffolds were washed three times with sterile PBS, and any remaining membrane from the transwell insert was carefully removed from the scaffolds, using sterile forceps. For each of the conditions, 6 scaffolds were harvested at each timepoint. Four of the 6 scaffolds were halved by weight for biochemical and gene expression analysis, while 2 were left whole for histological analysis. 

For biochemical analyses, each agarose scaffold half (*n* = 4) was digested in papain to release glycosaminoglycans and DNA. In brief, a digestion buffer consisting of 0.01 M cysteine hydrochloride (BDH, Dorset, UK), 0.05 M ethylenediaminetetraacetic acid (EDTA; Sigma-Aldrich) and 0.2 M sodium acetate (BDH) was prepared in distilled water and adjusted to pH6, using 1 M sodium hydroxide in distilled water (Sigma-Aldrich). Papain (Sigma-Aldrich) was then added to the digestion buffer to reach a final concentration of 125 µg/mL. Each half scaffold was digested in 1 mL of papain for 16 h at 65 °C, with regular vortexing, followed by 70 °C for 10 min to melt any residual agarose [28,29,30,31]. The resulting suspension was then immediately centrifuged at 10,000 rpm for 5 min to pellet any remaining fragments of agarose and other impurities, before being stored at −20 °C for future use [32]. 

DNA was quantified by using the PicoGreen^®^ fluorescence assay (Invitrogen, Loughborough, UK) as per the manufacturer’s instructions. Samples were assessed in duplicate, and unknown concentrations were interpolated from a standard curve, multiplied by the dilution factor (9), and then by two, to account for the whole scaffold, giving a final value of the total scaffold DNA content in ng. Cell number was calculated by using the widely accepted value of 7.7 pg of DNA per chondrocyte [33], and a mean cell number for each condition at each timepoint was calculated. 

The dimethylmethylene blue (DMMB) assay was used to quantify GAG content in the papain-digested agarose scaffolds (*n* = 4) [34,35]. Samples were assessed in duplicate, and total GAG values for samples were calculated by using the equation of the resulting standard curve. The resulting values were multiplied by two, to account for the whole scaffold, to give a final value of the total GAG in the scaffold in µg. A mean total GAG value for each condition at each timepoint was calculated. Mean total GAG values and cell numbers were used to calculate a mean GAG/cell value for each condition at each timepoint.

The remaining 2 scaffolds were processed by using an adapted protocol for the histological preparation of various three-dimensional culture systems [28]. In brief, scaffolds were fixed in paraformaldehyde overnight before being dehydrated through a series of gradually increasing concentrations of isopropyl alcohol (IPA) (30%, 50%, 70%, 80% 100% x2; 30 min each), cleared in Xylene (Genta Medical, York, UK) and embedded in paraffin wax (Sigma-Aldrich). Sections were then cut at a thickness of 8 µm and stained by using haematoxylin and eosin (H&E) and Alcian Blue to determine cell distribution and GAG deposition, respectively. 

For gene-expression analysis, each scaffold half (*n* = 4) was first dissolved in 1 mL of dissolution buffer from a stock solution made up with 20 mL Buffer RLT (Qiagen, Manchester, UK), 15 mL Buffer QG (Qiagen) and 1% (*v*/*v*) β-mercaptoethanol (Sigma-Aldrich) in 1.5 mL Eppendorf tubes [36]. Upon the addition of the buffer mix, the samples were left at room temperature until complete dissolution and then stored at −20 °C until ready to use. After defrosting, the samples were homogenised by vortexing, and 1 mL of 70% ethanol (molecular biology grade; Sigma-Aldrich) in RNAse free water was added. RNA was extracted by using the RNeasy^®^ Mini Kit (Qiagen) and by following the manufacturer’s instructions. Complementary DNA (cDNA) was generated from the extracted RNA by reverse transcription, using the High-Capacity cDNA Reverse Transcription Kit (Applied Biosystems, Loughborough, UK) according to the manufacturer’s instructions. Then qRT-PCR was performed on the Quant Studio 3 Real-Time Quantitative PCR System (Applied Biosystems), using SYBR™ green QuantiTect primer assays (Qiagen) to assess a number of genes associated with chondrocyte health and extracellular matrix production: SRY-Box Transcription Factor 9 (SOX9), Collagen type 2 alpha 1 (*COL2A1*), aggrecan (*ACAN*), collagen type X (*COL10A1*) and activin receptor-like kinase 1 (*ALK1*), as well as genes associated with extracellular matrix breakdown; matrix metalloproteinase (*MMP*) -3 and -13 and A disintegrin and metalloproteinase with thrombospondin motifs (*ADAMTS*) -4 and -5. Glyceraldehyde-3-phosphate dehydrogenase (*GAPDH*) and hypoxanthine phosphoribosyltransferase 1 (*HPRT1*) were employed as housekeeping genes. Then qRT-PCR was carried out according to the manufacturer’s instructions. Cycle threshold (Ct) values were collected at the end of the extension stage of each cycle. Sample gene expression levels were normalised to the geometric mean of their corresponding housekeeping genes [37]. Following normalisation to the reference genes, the relative expression of each gene at day 21 compared to day 0 was determined, using the ΔΔCt method and this was compared between early OA and late-OA conditions [38]. A twofold change (upregulated or downregulated) was deemed biologically significant.

#### 2.3.5. Endpoint Analysis—Conditioned Media 

For each patient, at each endpoint, conditioned media was pooled from experimental repeat wells, collected and labelled as 1 mL aliquots and stored at −20 °C until required. 

ELISAs were used to assess the abundance of several proteins of interest in the conditioned media, collected from the two co-culture conditions (early OA BM-MSCs and late-OA BM-MSCs) and chondrocyte only controls at day 0, day 7 and day 21. Matrix metalloproteinase-13 (MMP-13) was assessed by using a human Quantikine^®^ ELISA kit (R&D Systems, Minneapolis, MN, USA). Matrix metalloproteinase-1 (MMP-1), transforming growth factor-β1 (TGF-β1) and vascular endothelial growth factor (VEGF) were assessed by using duo-set ELISAs (R&D Systems). All ELISAs were carried out by using neat, conditioned media samples and in accordance with the manufacturer’s instructions. In all cases, samples were assessed in duplicate, and the mean optical density values were calculated and corrected for background interference and the average zero standard optical density. GraphPad Prism software, version 6.0 (GraphPad Software, San Diego, CA, USA) was used to plot dose-response standard curves of the log of the known concentration values against the optical density, which was then used to interpolate unknown values. For all analytes, the detection limit (DL) was determined [39]. The DL was based on the standard deviation of the response, and the slope and can be expressed as follows:

$$DL=\frac{3.3\sigma }{S}$$

For all analyses, values below the DL were replaced with the DL value as is common practice.

### 2.4. Statistical Analyses 

For all analyses, statistical significance was considered at *p* < 0.05. The distribution of data was checked by using the Shapiro–Wilk normality test. Normally distributed, continuous data were summarised as means and standard errors of the mean (mean ± SEM). 

#### 2.4.1. qRT-PCR Data

The relative expression of each of the genes of interest to the geometric mean of their corresponding housekeeping genes was calculated by using the ΔCt method [37,38]. The relative expression at day 0, to that at day 21, was calculated (ΔΔCt) [38]:

ΔΔCt = ((Ct gene of interest − Ct housekeeping genes) Day 0 − (Ct gene of interest − Ct housekeeping genes) Day 21)

Finally, fold change was calculated by using the comparative Ct method (2^−^^ΔΔCt^) [38]. Fold change was compared between conditions, using an independent samples *t*-test in Jamovi (version 1.2.8, jamovi.org, Sydney, Australia).

#### 2.4.2. ELISA Data

Linear mixed modelling was used to assess the ELISA data, for each of the analytes separately, using a random intercept and BM-MSC type and time point as fixed factors. Post hoc Bonferroni tests were used to carry out multiple comparisons to interrogate individual relationships between BM-MSC types and timepoints for each analyte.

#### 2.4.3. Biochemistry Data 

Linear mixed modelling was used to assess the biochemistry data (DNA, GAG and GAG/cell) to determine differences between conditions. Post hoc Bonferroni tests were used to carry out multiple comparisons to interrogate individual relationships between BM-MSC types and timepoints.

#### 2.4.4. Statistical Software

All data were organised by using Microsoft^®^ Office Excel 2013 (Microsoft^®^, Redmond, WA, USA). For all analyses, data from the five patients were combined, and comparisons were carried out between conditions and between timepoints, not between patients. Statistical analyses were carried out by using jamovi (version 1.2.8, jamovi.org), a free software interface that is built upon the R statistical package [40,41].

## 3. Results

### 3.1. Macroscopic and Histological Assessment of Tissue

**Figure 4:**
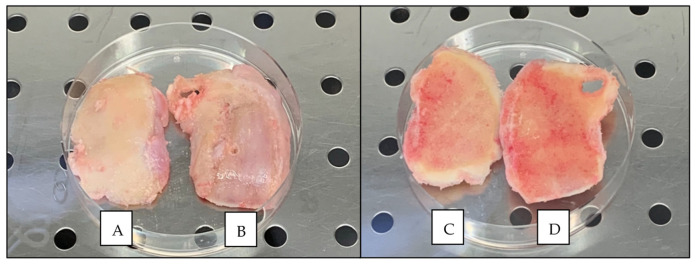
Example of the macroscopic appearance of knee condyles collected from one patient following TKR surgery. (**A**) Condyle with the best (lowest) ICRS graded cartilage (cartilage side). (**B**) Condyle with the worst (highest) ICRS graded cartilage (cartilage side). (**C**) Condyle with the best ICRS graded cartilage (bone side). (**D**) Condyle with the worst (highest) ICRS graded cartilage (bone side). Note that cartilage is eroded down to the bone in the worst graded condyle (**B**).

The cartilage of the excised tissue segments from TKR ranged in ICRS grade from grade 1, which is ‘nearly normal’, to ICRS grade 4, which is ‘severely abnormal’, with a median ICRS grade of 2 (Figure 4, Table 1). The tissue that demonstrated the best and worst ICRS grade were collected from each patient, resulting in 10 tissue segments in total from TKR patients. Subsequently, the macroscopically graded segments were sectioned and stained for histological scoring. The cartilage ranged in OARSI histopathology grade from grade 1, which is ‘surface intact with superficial fibrillation’, to grade 5, which is ‘denudation’, with a median OARSI grade of 3.25. The SB ranged in histological grade from grade 0, which is ‘early stages of OA’, to grade 3, which is ‘late-stage disease’, and the median SB histological grade was 0 (Table 1). 

**Figure 5:**
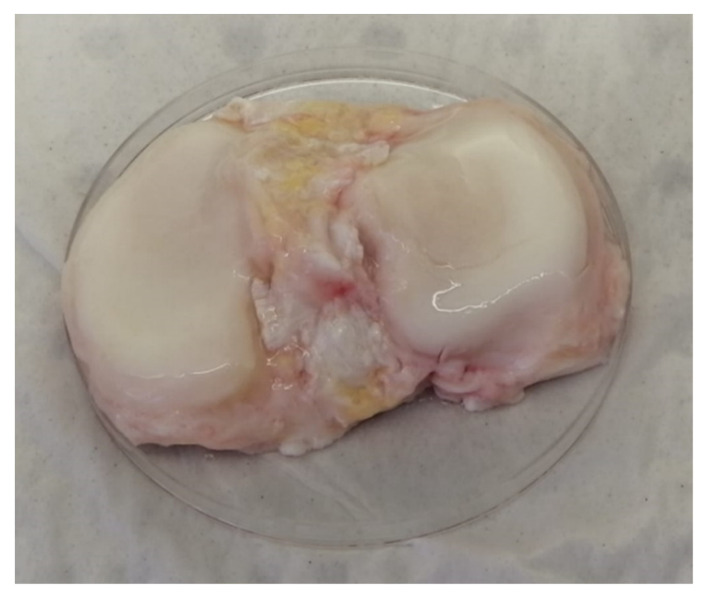
Macroscopic appearance of the tibial plateau obtained from a young patient with no history of OA, sourced from the NHSBT. Right knee from posteroanterior view.

The tibial plateau obtained from the young, healthy patient demonstrated ICRS grade 0 ‘normal’ cartilage with a smooth, glassy surface and no evidence of fibrillation, indentations or lesions (Figure 5). The AC of the healthy sample was white in colour and did not demonstrate any yellowing typically associated with ageing in cartilage and which was observed in the cartilage of all condylar samples obtained from TKR. Stained sections of cartilage obtained from the young patient demonstrated an OARSI cartilage histopathology grade of 0, ‘normal’ cartilage.

### 3.2. Chondrocyte–Agarose Scaffolds 

**Figure 6:**
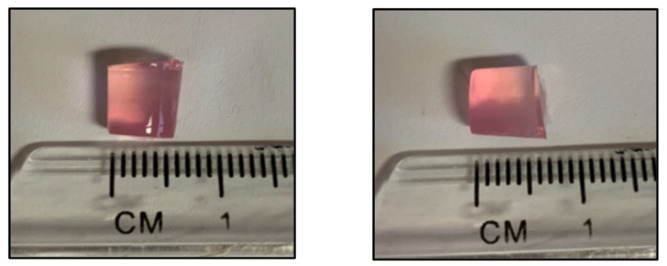
Representative images of a chondrocyte-agarose scaffold removed from the 24-well inserts in the co-culture model for analysis.

Agarose–chondrocyte scaffolds were collected from the early OA and late-OA co-cultures and from the chondrocyte-only control at each of the four timepoints. The scaffolds that resulted from the co-culture were cylindrical in shape, had a diameter of roughly 6 mm and a height of roughly 6 mm (Figure 6). There was no difference in the mean weight of the scaffolds between any of the conditions at any of the four timepoints.

#### 3.2.1. Histological Analysis

**Figure 7:**
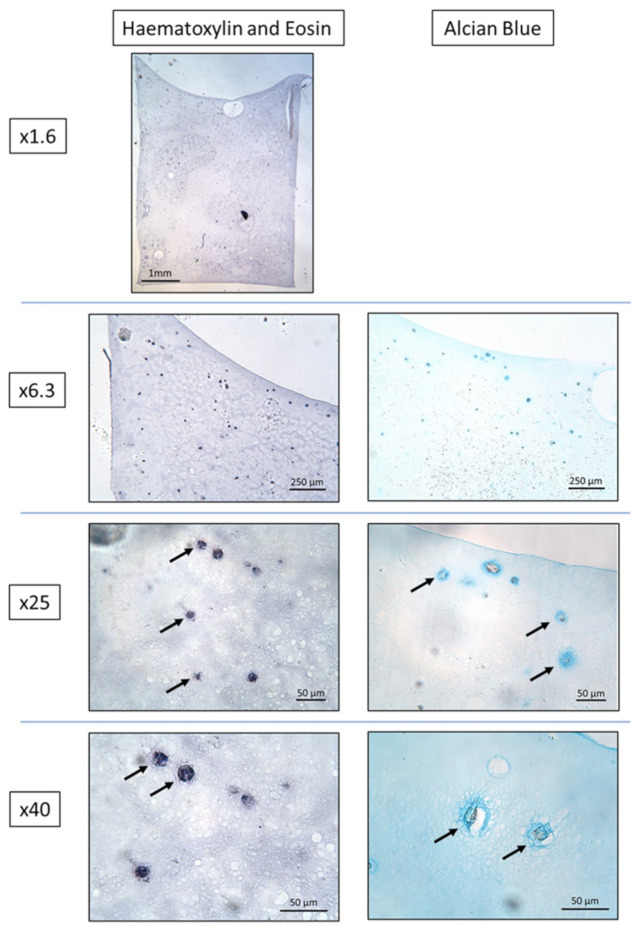
Representative images of H&E and Alcian Blue–stained sections from the day 21 chondrocyte-loaded agarose scaffolds co-cultured with early OA BM-MSCs. Solid arrows: chondrocytes. Note the Alcian Blue staining surrounding the chondrocytes, indicating GAG deposition.

H&E staining of the chondrocyte-loaded agarose scaffolds at day 0 and at day 21 demonstrated that the chondrocytes were well-distributed throughout the scaffolds in all conditions. Alcian Blue staining revealed no evidence of GAG deposition in any of the conditions at day 0, as was expected. At day 21 GAG staining was present in a circular pattern immediately surrounding the chondrocytes in all conditions (Figure 7). There were no obvious differences in either H&E or Alcian Blue staining between the three conditions at day 0 nor at day 21.

#### 3.2.2. Chondrocyte Number 

**Figure 8:**
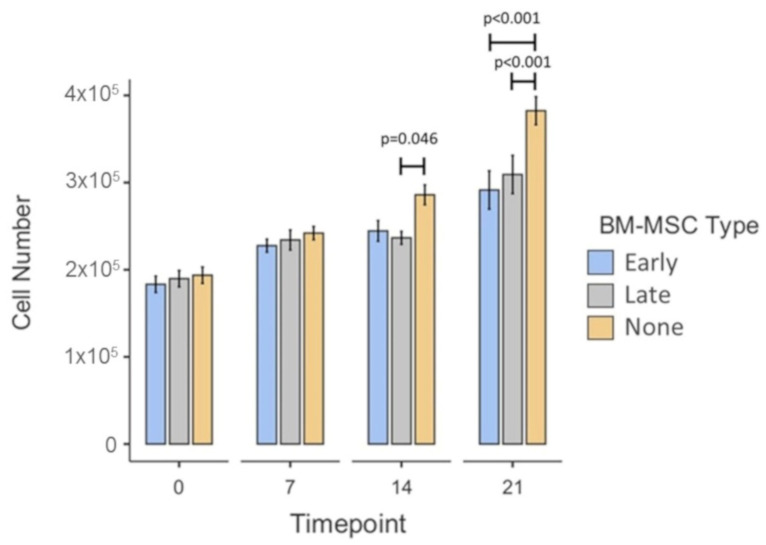
Mean chondrocyte number in scaffolds taken from the co-culture model at the four timepoints. Error bars represent the standard error of the mean. Significant differences are indicated by horizontal bars.

The mean chondrocyte number in the co-culture model across all three conditions increased over time, with a significant increase between day 0 (1.89 × 10^5^ ± 5.36 × 10^3^_SEM_) and day 7 (2.34 × 10^5^ ± 5.21 × 10^3^_SEM_; *p* < 0.001), between day 7 and day 14 (2.55 × 10^5^ ± 6.5 × 10^3^_SEM_; *p* = 0.024) and between day 14 and day 21 (3.28 × 10^5^ ± 1.25 × 10^4^_SEM_; *p* < 0.001) (Figure 8). Post hoc testing provided more detailed comparisons between conditions at individual timepoints. There were no significant differences between any of the conditions at day 0 nor at day 7. At day 14, scaffolds taken from co-cultures with late-OA BM-MSCs contained significantly lower mean chondrocyte numbers (2.37 × 10^5^ ± 7.38 × 10^3^_SEM_) than those taken from the chondrocyte-only co-cultures (2.86 × 10^5^ ± 1.13 × 10^4^_SEM_; *p* = 0.046). At day 21, the mean chondrocyte number in scaffolds taken from the chondrocyte-only condition (3.82 × 10^5^ ± 1.61 × 10^4^_SEM_) was significantly greater than in those taken from both the early OA (2.91 × 10^5^ ± 2.19 × 10^4^_SEM_; *p* < 0.001) and late-OA (3.09 × 10^5^ ± 2.19 × 10^4^_SEM_; *p* < 0.001) co-cultures. There was no significant difference at this timepoint between the early OA and late-OA co-culture conditions (*p* = 1.00).

#### 3.2.3. Total GAG

**Figure 9:**
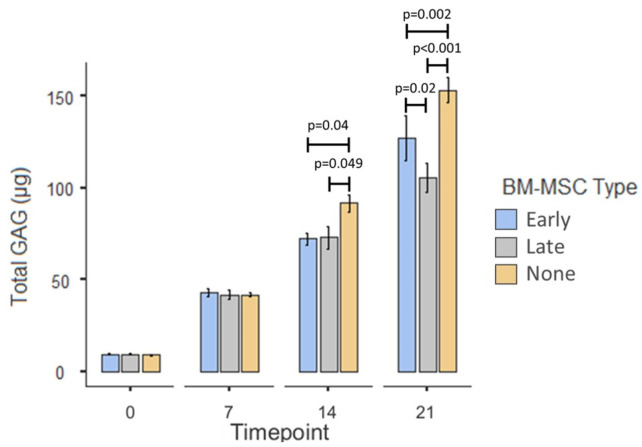
Mean total GAG content of agarose scaffolds taken from the co-culture model at the four timepoints. Error bars represent the standard error of the mean. Significant differences are indicated by horizontal bars.

The mean GAG content of the scaffolds, across all conditions, increased over time with significant differences between day 0 (9.09 µg ± 0.16_SEM_) and day 7 (42.1 µg ± 1.09_SEM_; *p* < 0.001), between day 7 and day 14 (78.7 µg ± 2.94_SEM_; *p* < 0.001) and between day 14 and day 21 (129 µg ± 5.83_SEM_; *p* < 0.001; Figure 9). Post hoc testing revealed that there were no significant differences between any of the conditions at day 0 or at day 7. At day 14 the mean total GAG content of the chondrocyte-only control scaffolds (91.5 µg ± 4.41_SEM_) was significantly higher than that in scaffolds from both the early OA co-cultures (72.2 µg ± 3.21_SEM_; *p* = 0.04) and the late-OA co-cultures (72.9 µg ± 6.13_SEM_; *p* = 0.049). At day 21, the mean total GAG content of the chondrocyte-only control scaffolds (153 µg ± 6.65_SEM_) was significantly higher than those from the early OA co-culture (127 µg ± 12.2_SEM_; *p* = 0.002) and the late-OA co-culture (106 µg ± 7.88_SEM_; *p* < 0.001). Moreover, the mean total GAG content of the early OA co-culture scaffolds was significantly higher than that of the late-OA co-culture scaffolds (*p* = 0.02).

#### 3.2.4. GAG per Chondrocyte

**Figure 10:**
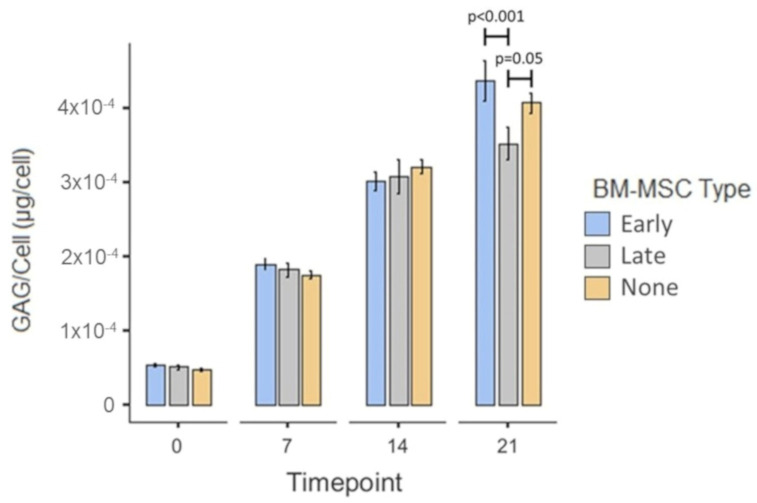
Mean GAG/chondrocyte of the scaffolds taken from the co-culture model at the four timepoints. Error bars represent the standard error of the mean. Significant differences are indicated by horizontal bars.

The mean GAG/chondrocyte across all conditions increased over time with a significant increase between day 0 (5.02 × 10^−5^ µg/cell ± 1.55 × 10^−6^_SEM_) and day 7 (1.82 × 10^−4^ µg/cell ± 4.3910^−6^_SEM_; *p* < 0.001), between day 7 and day 14 (3.09 × 10^−4^ µg/cell ± 9.14 × 10^−6^_SEM_; *p* < 0.001) and between day 14 and day 21 (3.99 × 10^−4^ µg/cell ± 1.30 × 10^−5^_SEM_; *p* < 0.001; Figure 10). There was no significant difference in mean GAG/chondrocyte between conditions at day 0, day 7 nor day 14 (all *p* = 1.00). At day 21 the average GAG/chondrocyte was significantly higher in the scaffolds from the early OA co-culture (4.37 × 10^−4^ µg/cell ± 2.69 × 10^−5^_SEM_) than in those from the late-OA co-culture (3.52 × 10^−4^ µg/cell ± 2.19 × 10^−5^_SEM_; *p* < 0.001). The average GAG/chondrocyte in the chondrocyte-only scaffolds was borderline significantly higher (4.07 × 10^−4^ µg/cell ± 1.36 × 10^−5^_SEM_; *p* = 0.05) than in the late-OA BM-MSC co-cultured scaffolds, but there was no significant difference between the chondrocyte-only scaffolds and the scaffolds co-cultured with early OA BM-MSCs (*p* = 1.00).

#### 3.2.5. Gene Expression Analysis

We used qRT-PCR to assess the relative expression of a number of genes in the chondrocytes encapsulated in agarose over the course of the co-culture model (day 21 vs. day 0). The relative gene expression was then compared between conditions to assess the effect of early OA and late-OA BM-MSCs, as well as no BM-MSCs (chondrocyte-only control) on the gene expression profile of the agarose-encapsulated chondrocytes. 

**Figure 11:**
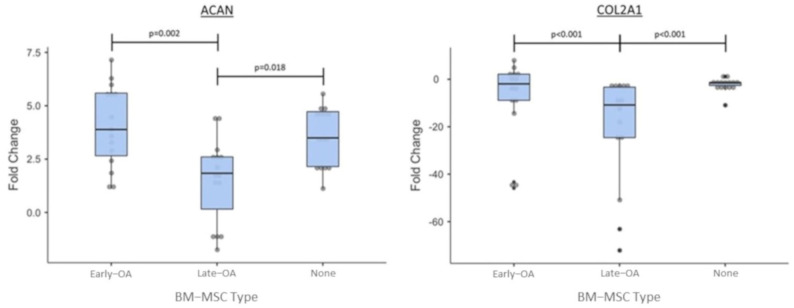
Relative fold change of *ACAN* and *COL2A1* between day 0 and day 21 of the co-culture. Error bars represent the standard error of the mean. Significant differences are indicated by horizontal bars.

**Figure 12:**
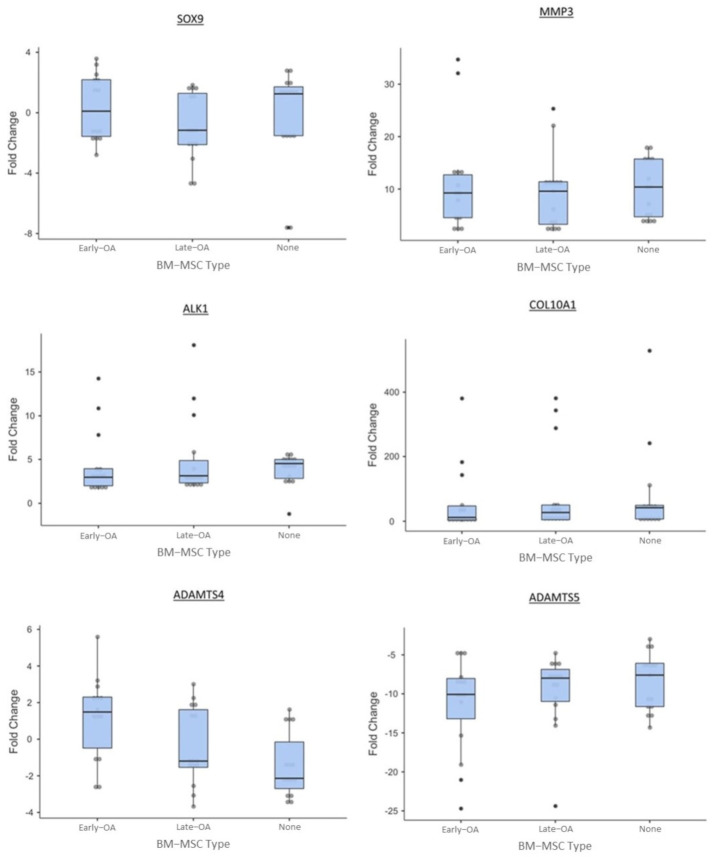
Relative fold change of additional genes of interest between day 0 and day 21 of the co-culture. Error bars represent the standard error of the mean. Significant differences are indicated by horizontal bars.

For the majority of genes, a consistent pattern of expression was demonstrated in the chondrocytes recovered from all three culture conditions. All conditions demonstrated a biologically significant downregulation of *COL2A1* between day 0 and day 21. There was a significantly greater downregulation of *COL2A1* in the late-OA condition (−32.8 ± 12.9_SEM_) compared to both the early OA (−7.82 ± 4.46_SEM_; *p* < 0.001) and chondrocyte-only (−2.32 ± 0.74_SEM_; *p* < 0.001) conditions (Figure 11). There was no significant difference in relative expression of *COL2A1* between the early OA and the chondrocyte-only control (*p* = 0.069). A biologically significant upregulation of *ACAN* from day 0 to day 21 was observed in all conditions. The chondrocytes from the co-culture with late-OA BM-MSCs (1.51 ± 0.51_SEM_) showed a significantly lower upregulation of *ACAN* between day 0 and day 21 than both the early OA (4.05 ± 0.49_SEM_, *p* = 0.002) and chondrocyte-only (3.57 ± 0.36_SEM_; *p* = 0.018) conditions (Figure 11). There was no significant difference in *ACAN* fold change between the scaffolds from the early OA co-culture and those from the chondrocyte-only control condition (*p* = 0.624; Figure 11). There was no biologically significant fold change in *ADAMTS-4* or *SOX9* expression over the course of the study in any of the three conditions (Figure 12). All conditions showed a biologically significant upregulation of *ALK-1, COL10A1, MMP-3* and *MMP-13* and downregulation of *ADAMTS-5* over the course of the study, but there were no significant differences between conditions in the expression of any of these genes (Figure 12). 

### 3.3. Conditioned Media 

At each timepoint, conditioned media was collected from the 24-well plate and pooled for each condition (early OA, late OA and chondrocyte-only control).

**Figure 13:**
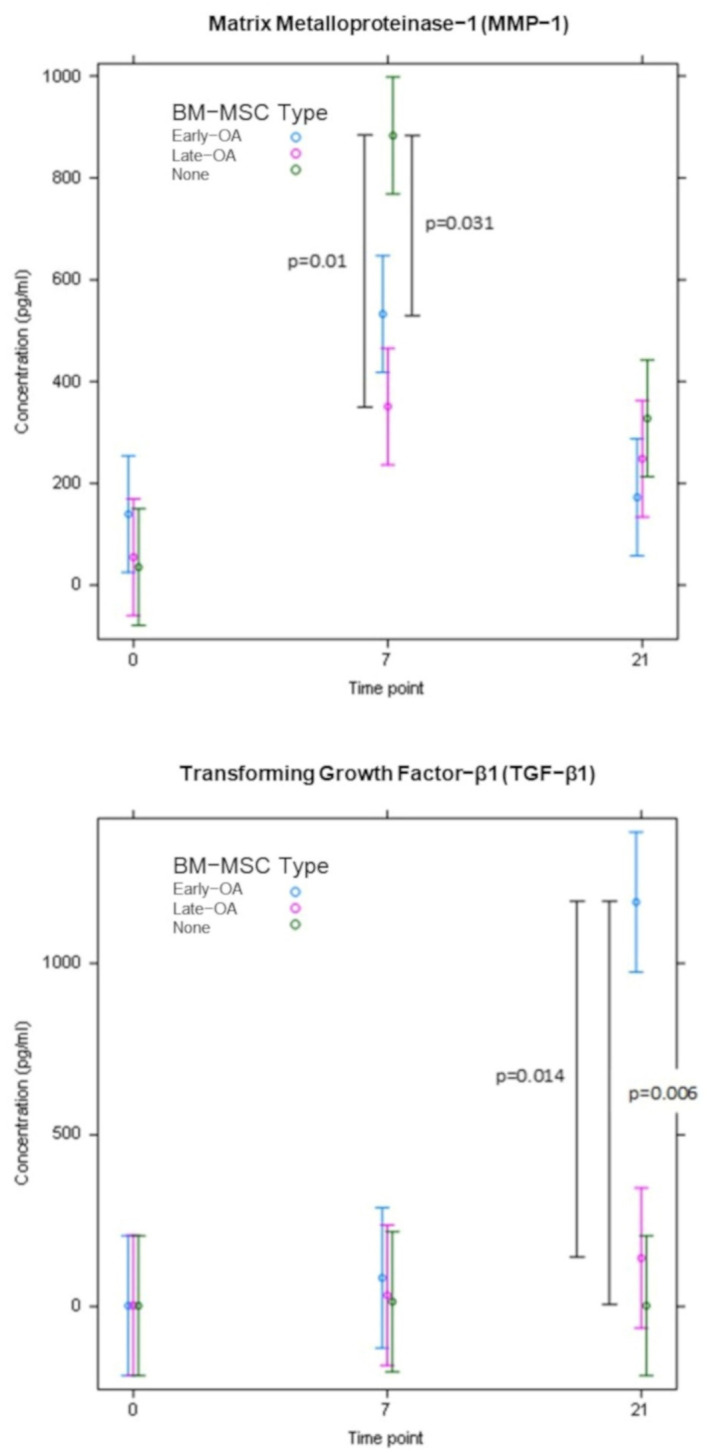
The concentration of MMP-1 and TGF-β1 in conditioned media at day 0, day 7 and day 21 of the co-culture model, as determined by ELISA. Error bars represent the standard error of the mean. Significant differences are indicated by vertical black bars.

**Figure 14:**
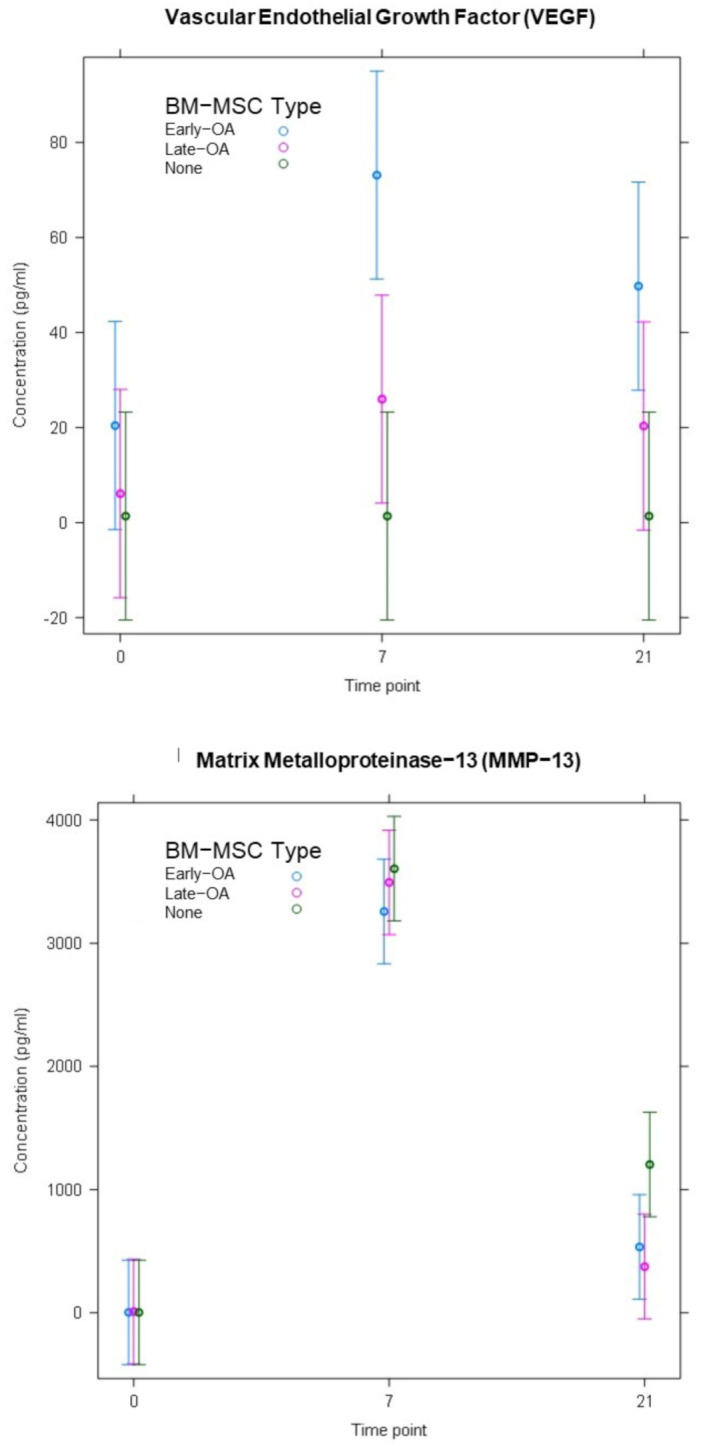
The concentration of VEGF and MMP13 in conditioned media at day 0, day 7 and day 21 of the co-culture model, as determined by ELISA. Error bars represent the standard error of the mean. Significant differences are indicated by vertical black bars.

ELISAs were carried out on conditioned media collected at day 0, day 7 and day 21 from co-cultures containing early OA and late-OA BM-MSCs as well as the chondrocyte-only control (no BM-MSCs). The concentration of MMP-1 (DL, 3.2697 pg/mL; Figure 13) and MMP-13 (DL, 2.7924 pg/mL; Figure 14) in the conditioned media demonstrated similar patterns over the course of the study across all conditions, with a significant increase between day 0 and day 7 and a decrease between day 7 and day 21. There were, however, no significant differences between conditions in MMP-13 concentration at any of the timepoints. There were no significant differences in MMP-1 concentration between any of the conditions at day 0 or at day 21, but at day 7, the mean concentration of MMP-1 in the conditioned media from the chondrocyte only control (883 pg/mL ± 260_SEM_) was significantly higher than in the conditioned media from both the early OA (532 pg/mL ± 116_SEM_, *p* = 0.031) and late-OA (351 pg/mL ± 119_SEM_, *p* = 0.01) BM-MSC co-cultures (Figure 13). There were no significant differences between timepoints or between conditions in the concentration of VEGF (DL, 1.6963; Figure 14). The concentration of TGF-β1 (DL: 1.3693) was very low in all conditions at day 0 and day 7, with majority of conditioned media samples were below the detection limit and therefore were replaced with the non-detect value (1.7 pg/mL) (Figure 13). At day 0 and day 7, there was no significant difference in mean TGF-β1 concentration in conditioned media between conditions (all *p* = 1.00). At day 21, there was a significant difference between the mean concentration of TGF-β1 in conditioned media taken from the early OA BM-MSC co-culture (1178 pg/mL ± 589_SEM_) and that taken from the late-OA BM-MSC co-culture (141 pg/mL ± 7.22_SEM_; *p* = 0.014) and from the chondrocyte-only control (1.7 pg/mL ± 0_SEM_; *p* = 0.006). Between day 7 and day 21, conditioned media collected from the co-culture with early OA BM-MSCs demonstrated a significant increase in mean TGF-β1 concentration (day 7, 83.1 pg/mL ± 81.4_SEM_; day 21, 1178 pg/mL ± 589_SEM_; *p* = 0.018; Figure 13).

## 4. Discussion

We demonstrated that the presence of BM-MSCs in the model, whether isolated from early OA or late-OA tissue, inhibited the proliferation and overall GAG production of the agarose-encapsulated, healthy chondrocytes. Proliferation and GAG deposition were evident in all conditions over the course of the experiment but at day 14 and day 21, both were significantly higher in the chondrocyte-only control (no BM-MSCs) than in either of the co-culture conditions. One possible explanation could be the effect of mediators produced by the BM-MSCs. It is well-known that MSCs are able to secrete trophic factors that can exert modulating effects on chondrocytes, and this forms part of the basis for the use of MSCs in tissue repair. Xu and colleagues reported that indirect co-culture of rabbit articular chondrocytes with rabbit MSCs reduced proliferation of chondrocytes and production of cartilage ECM proteins, including GAGs, compared to monoculture of articular chondrocytes, and that this effect was mediated entirely via secreted factors [42]. The identity of these secreted factors in the rabbit study and our study is unknown and further research is required.

More relevant to our hypothesis, we demonstrated that co-culture with late-OA BM-MSCs led to a reduction in total GAG compared to co-culture with early OA BM-MSCs, as was evidenced by a significant difference in total GAG between the conditions at day 21. As there was no significant difference in chondrocyte number between the two conditions at any timepoint, we suspected that the difference in total GAG was caused by a difference in production per cell, rather than a difference in cell number. This was borne out when the total GAG was normalised to cell number, revealing a significant difference in GAG/cell between the early OA and late-OA conditions at day 21 of the co-culture. This finding demonstrates that the health state of BM-MSCs can influence the metabolic activity of healthy chondrocytes in indirect co-culture, suggesting that the effect is mediated entirely via soluble factors. This represents a key finding of the study, indicating that the biochemical composition of the repair cartilage is influenced by the health state of the SB. It is well established that the unique biomechanical properties of the AC are determined by its composition, and therefore a different composition of repair cartilage above bone demonstrating different stages of degeneration could lead to altered properties and altered outcome of the repair surgery. Interestingly, there was no significant difference in GAG/cell between the early OA co-culture condition and the chondrocyte-only control, indicating that the difference in total GAG between these conditions was due to a difference in chondrocyte number rather than a difference in the metabolic activity of the chondrocytes. This also suggests that late-OA BM-MSCs have an inhibitory effect on GAG metabolism, rather than early OA BM-MSCs having a stimulatory effect. Westacott and colleagues similarly reported that the metabolism of explanted cartilage, quantified by GAG secretion, was modulated by the health of subchondral bone cells [19]. These authors found that co-culture of cartilage explants with non-arthritic bone cells had no effect on GAG secretion, whereas in half of those that were co-cultured with bone cells from osteoarthritic bone, there was an increase in GAG secretion. Although this study is the most similar published work to our own, there are a number of methodological differences between our study and theirs, namely the use of osteoblasts rather than BM-MSCs and OA chondrocytes in place of those from a healthy donor. In addition, their study was carried out by using only three patients, all of whom were male. Therefore, comparisons between findings are useful, but they are drawn with caution throughout.

Gene expression analysis of the agarose-encapsulated chondrocytes revealed further interesting differences between culture conditions. The relative expression of *ACAN* in the chondrocytes that were co-cultured with early OA BM-MSCs mirrored that of the chondrocyte-only control, demonstrating a similar upregulation over the course of the study, with no significant difference between these conditions. However, there was no biologically significant change in *ACAN* expression in the chondrocytes that were co-cultured with late-OA BM-MSCs. Although there is not direct correlation between gene expression and corresponding protein levels, this finding goes some way to explain the reduced GAG/cell in the late-OA co-culture, as the *ACAN* gene encodes the aggrecan protein, a GAG that is highly abundant in the cartilage ECM. Work by Sanchez and colleagues demonstrated similar results, showing that *ACAN* expression was inhibited in OA chondrocytes which were co-cultured with sclerotic osteoblasts, compared to those cultured with non-sclerotic osteoblasts, and that the effect was mediated by soluble factors [20]. 

The relative expression of *COL2A1*, which encodes another of the major proteins of the ECM, collagen type 2, was also found to differ between chondrocytes cultured with early OA and late-OA BM-MSCs. Over the course of the study, there was an almost 33-fold downregulation in *COL2A1* expression in the chondrocytes that were cultured with late-OA BM-MSCs (fold change = −32.8 ± 12.9). This was significantly different to both the early OA BM-MSC co culture (*p* < 0.001) and the chondrocyte-only control (*p* < 0.001). There was also a downregulation of *COL2A1* in the chondrocytes from the early OA co-culture (fold change = −7.82 ± 4.46), and in the chondrocyte-only control (fold change = −2.32 ± 0.74), but these were not significantly different from one another. A similar pattern of *COL2A1* expression in co-cultured chondrocytes was reported by Sanchez and colleagues, who demonstrated that the indirect co-culture of OA chondrocytes with either sclerotic or non-sclerotic osteoblasts caused a downregulation of *COL2A1* in both conditions after four days, but that the downregulation was significantly greater in the sclerotic condition [21]. They also showed that after 10 days, *COL2A1* expression was only downregulated in the co-culture with sclerotic osteoblasts [21]. 

We also quantified the expression of a number of genes that encode matrix degrading enzymes to investigate whether co-culture with late-OA BM-MSCs caused an increase in the catabolism of matrix components as well as a decrease in anabolism. The *ADAMTS4* and *ADAMTS5* genes encode two of the major aggrecan degrading enzymes and MMP-3 and MMP-13 two of the major collagen degrading enzymes. There were no significant differences in expression between conditions in any of these genes over the course of the study. This suggests that decreased anabolic activity, rather than increased catabolic activity, inhibited GAG/cell production during co-culture with late-OA BM-MSCs, compared to co-culture with early OA BM-MSCs. This is demonstrated by the differential regulation of genes that encode matrix proteins, but not of genes that encode matrix degrading enzymes. 

We sought to identify soluble mediators of the communication between the BM-MSCs and the chondrocytes by assessing the concentration of five potentially relevant proteins in the conditioned media from the two co-culture conditions and the chondrocyte-only control. At day 21, the concentration of TGF-β1 in the conditioned media collected from the early OA co-culture condition (1178 ± 589 pg/mL) was significantly higher than in both the late-OA co-culture (141 ± 122; *p* = 0.014) and the chondrocyte-only control (*p* = 0.006), in which TGF-β1 was still undetectable. TGF-β is a potent inducer of cartilage ECM synthesis, as well as a suppressor of certain catabolic stimuli, found to be highly expressed in normal cartilage but absent in OA cartilage [43,44]. Therefore, we hypothesise that the differential secretion of TGF-β1 by the early OA and late-OA BM-MSCs could, in part, explain the differential expression of ECM matrix genes and the difference in GAG/cell in the co-cultured chondrocytes, but a further, knockout study would be required to establish this with certainty.

MSCs are known to have significant immunomodulatory capabilities which are suspected to underpin a significant proportion of their therapeutic efficacy [45]. These properties are partially mediated by the secretion of a range of bioactive substances, including growth factors, cytokines and chemokines [45,46,47]. Of the cytokines produced and secreted by MSCs, TGF-β is one of the most prominent. Besides promoting the remodelling of damaged tissues through the regulation of key cellular functions, TGF-β is also a principal regulator of immune function [45,46]. TGF-β exerts its immunomodulatory function in a number of ways, including the inhibition of T-cell proliferation, suppression of B cells and the generation of regulatory T cells [45,48]. Inflammation in the joint is a known risk factor for the progression of cartilage loss, and regulating the inflammatory landscape within the joint, part of the environment for tissue regeneration, is therefore increasingly being recognised as important in the treatment of OA [46,49]. Our study suggests that the immunomodulatory effect of TGF-β could also be locally mediated. Based on our findings, the differential secretion of TGF-β1 by early OA and late-OA BM-MSCs might not only drive the observed differences in chondrocyte metabolism between the two conditions directly, but also act to modify the inflammatory landscape in the model, thus providing different environments for repair. In order to test this theory, the inflammatory landscape of the joint environments simulated in this study must be characterised more fully by analysing additional pro- and anti-inflammatory cytokines in the conditioned media. The cause for differences in TGF-β1 secretion by MSCs from different locations in the same joint, the mechanism by which this differential secretion is maintained in culture-expanded cells, and the precise effect of TGF-β1 in an in vitro system such as ours are all yet to be elucidated.

Our study demonstrated that the co-culture of healthy chondrocytes with BM-MSCs isolated from regions with more advanced OA changes inhibited the expression of genes encoding the major ECM proteins and resulted in a decreased production of GAG. The application of these results, from the model to the clinical situation that it represents, suggests that the composition of repair cartilage that results from a cell-based therapy may vary depending on the health of the underlying SB. Thus, treatment of the SB prior to the implantation of cells, such as with the injection of BM-MSCs, as is described by Hernigou and colleagues, may result in a better repair cartilage [50,51]. However, we demonstrated that, even in the presence of BM-MSCs from the most degenerated regions of OA joints, cartilage still formed, suggesting that there is a possibility that an allogenic chondrocyte therapy may be a viable option for AC repair in patients with even severe OA. 

## 5. Conclusions

In summary, we have demonstrated that co-culture with late-OA BM-MSCs resulted in a reduction in GAG deposition and a downregulation of the genes encoding collagen type 2 and aggrecan in healthy articular chondrocytes, compared to culture with early OA BM-MSCs. We also identified TGF-β1 as a differentially secreted growth factor that could possibly mediate the differential effects of the two BM-MSC populations. Although this work was performed in vitro, there are clear implications for cartilage repair by cell-based tissue engineering, in which the health of the SB may influence the composition and, therefore, properties of the repair tissue. 

## Figures and Tables

**Table 1:** Demographic information, macroscopic (ICRS Cartilage Lesion Grade) and histological scores (OARSI OA Cartilage Histopathology Grade and Subchondral Bone Histological Grade) of condyles obtained from patients undergoing TKR surgery.

Patient	Gender	Age	Knee	Tissue Segment	ICRS Grade	OARSI Grade	SB Grade
1	Male	72	Left	Early OA	1	1	0
				Late OA	4	3.5	1
2	Female	70	Left	Early OA	1	1	0
				Late OA	4	5	2
3	Male	76	Right	Early OA	1	1	0
				Late OA	4	5	3
4	Female	73	Right	Early OA	1	2	0
				Late OA	4	5	2
5	Female	73	Left	Early OA	1	2	0
				Late OA	4	5	2
	Mean ± SD	72.8 ± 2.2					

## Data Availability

The raw data analysed in this study are available on request from the corresponding author.
